# Supplementary material for: A skin-permeable polymer for non-invasive transdermal insulin delivery
Source: Nature. 2025 Nov 19;648(8093):459–67. doi: 10.1038/s41586-025-09729-x (PMC12695667; doi:10.1038/s41586-025-09729-x)
Supplement: Supplementary file 1 — Supplementary Figs 1–32 and Supplementary Tables 1 and 2. [file 41586_2025_9729_MOESM1_ESM.pdf]

nature portfolio

https://doi.org/10.1038/s41586-025-09729-x

Supplementary information

A skin-permeable polymer for non-invasive transdermal insulin delivery

In the format provided by the authors and unedited

Supplementary Information

A skin-permeable polymer for noninvasive transdermal insulin delivery

Qiuyu Wei[1†], Zhi He[2†], Zifan Li[1†], Zhuxian Zhou[1†], Ying Piao[1], Jianxiang Huang[2], Yu Geng[1], Runnan Zhang[1], Yaqi Fu[2], Jiayi Ye[1], Yue Yuan[1], Haoru Zhu[1], Jiaheng Zeng[1], Yan Zhang[1], Quan Zhou[1], Mingyu Xu[2], Shiqun Shao[1], Jianbin Tang[1], Jiajia Xiang[1*], Rongjun Chen[3*], Ruhong Zhou[2*] & Youqing Shen[1*]

[1]Zhejiang Key Laboratory of Smart Biomaterials and Center for Bioengineering, Key Laboratory of Biomass Chemical Engineering of Ministry of Education, State Key Laboratory of Chemical Engineering, College of Chemical and Biological Engineering, Zhejiang University, Hangzhou, China 310058.

[2]Institute of Quantitative Biology, Zhejiang Key Laboratory of Cell and Molecular Intelligent Design and Development, College of Life Science, Zhejiang University, Hangzhou, China 310058.

[3]Department of Chemical Engineering, Imperial College London, South Kensington Campus, London SW7 2AZ, UK.

*Corresponding authors: Youqing Shen (shenyq@zju.edu.cn); Ruhong Zhou (rhzhou@zju.edu.cn); Rongjun Chen (rongjun.chen@imperial.ac.uk); Jiajia Xiang (xiang_jj@zju.edu.cn).

†These authors contributed equally to this work.

Contents

Supplementary Fig. 1 3
Supplementary Fig. 2 4
Supplementary Fig. 3 5
Supplementary Fig. 4 6
Supplementary Fig. 5 7
Supplementary Fig. 6 8
Supplementary Fig. 7 9
Supplementary Fig. 8 10
Supplementary Fig. 9 11
Supplementary Fig. 10 12
Supplementary Fig. 11 13
Supplementary Fig. 12 14
Supplementary Fig. 13 15
Supplementary Fig. 14 16
Supplementary Fig. 15 17
Supplementary Fig. 16 18
Supplementary Fig. 17 19
Supplementary Fig. 18 20
Supplementary Fig. 20 22
Supplementary Fig. 21 23
Supplementary Fig. 22 24
Supplementary Fig. 23 25
Supplementary Fig. 24 26
Supplementary Fig. 25 27
Supplementary Fig. 26 28
Supplementary Fig. 27 29
Supplementary Fig. 28 30
Supplementary Fig. 29 31
Supplementary Fig. 30 32
Supplementary Fig. 31 33
Supplementary Fig. 32 34
Supplementary Table 1 35
Supplementary Table 2 36

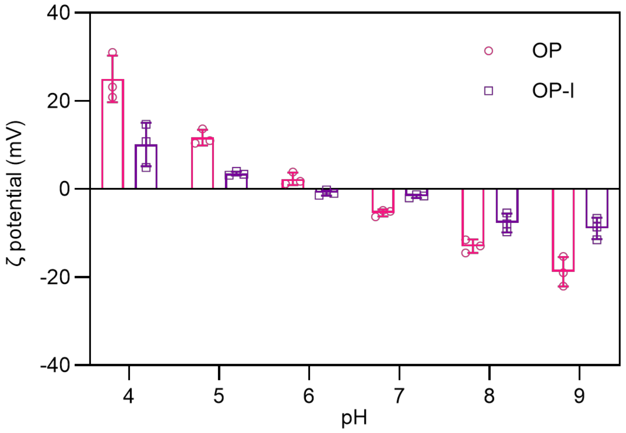

Supplementary Fig. 1 | The pH-dependent zeta-potentials of OP (0.1 mg/mL) and OP-I (0.04 mg/mL) in HEPES buffer at different pH. Data are presented as mean ± s.d.; *n* = 3 independent experiments.

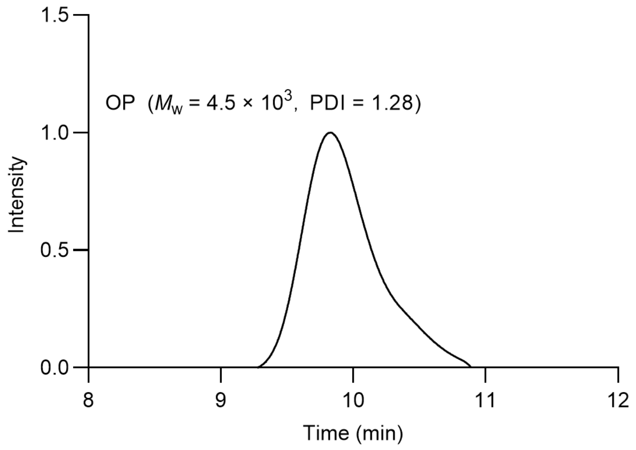

Supplementary Fig. 2 | The gel permeation chromatography (GPC) trace of OP.

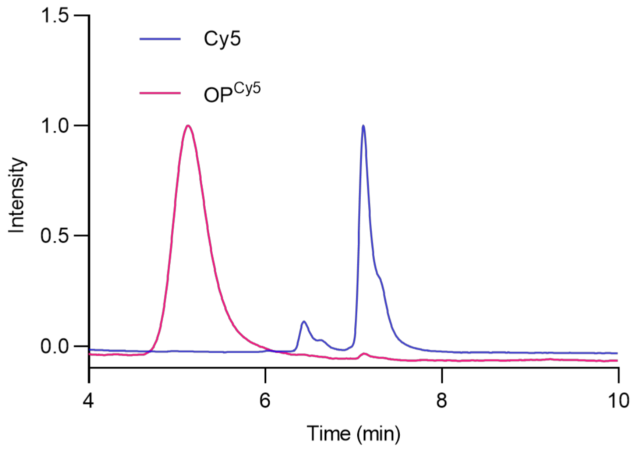

Supplementary Fig. 3 | The high-performance liquid chromatography (HPLC) traces of Cy5 and OP^{Cy5} with absorbance at 280 nm.

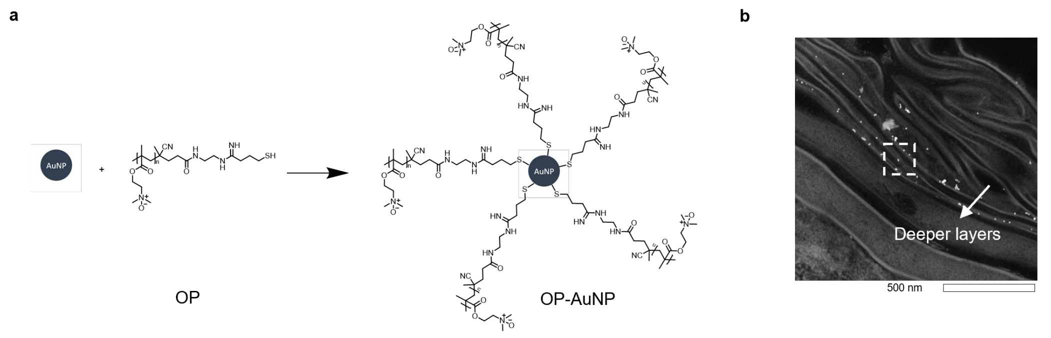

Supplementary Fig. 4 | Permeability of OP-AuNPs in the mouse stratum corneum (SC). a, Synthesis of OP-AuNPs. **b**, The HAADFI-STEM image of the SC of the mouse skin after 4 h of topical application with OP-AuNPs (OP-eq. dose: 0.2 mL of 0.5 mg/mL; application area: 1.13 cm^2). The image is representative of *n* = 3 independent experiments.

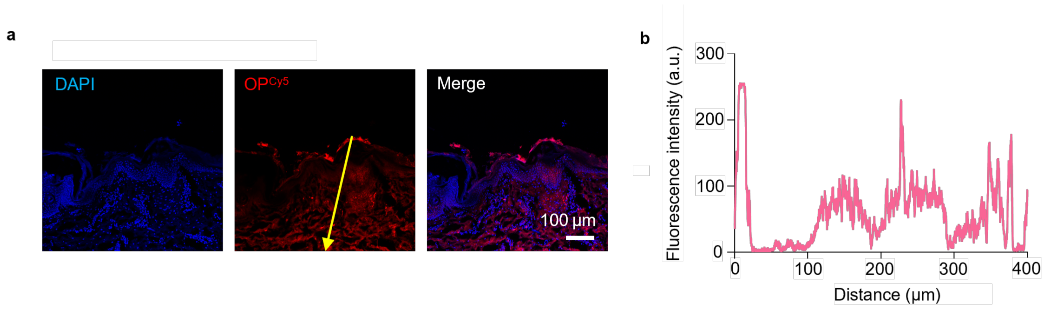

Supplementary Fig. 5 | Skin permeability of OP^{Cy5} in minipig. a, The confocal laser scanning microscope (CLSM) images of the minipig abdominal skin slices after topical application for 4 h with OP^{Cy5} (Cy5-eq. dose: 10 mL of 10 μg/mL; application area: 100 cm^2); **b**, Line-scan analysis of Cy5 fluorescence along a randomly selected line (yellow arrow in panel a) from the skin surface to the subcutis. The images are representative of n = 3 independent experiments.

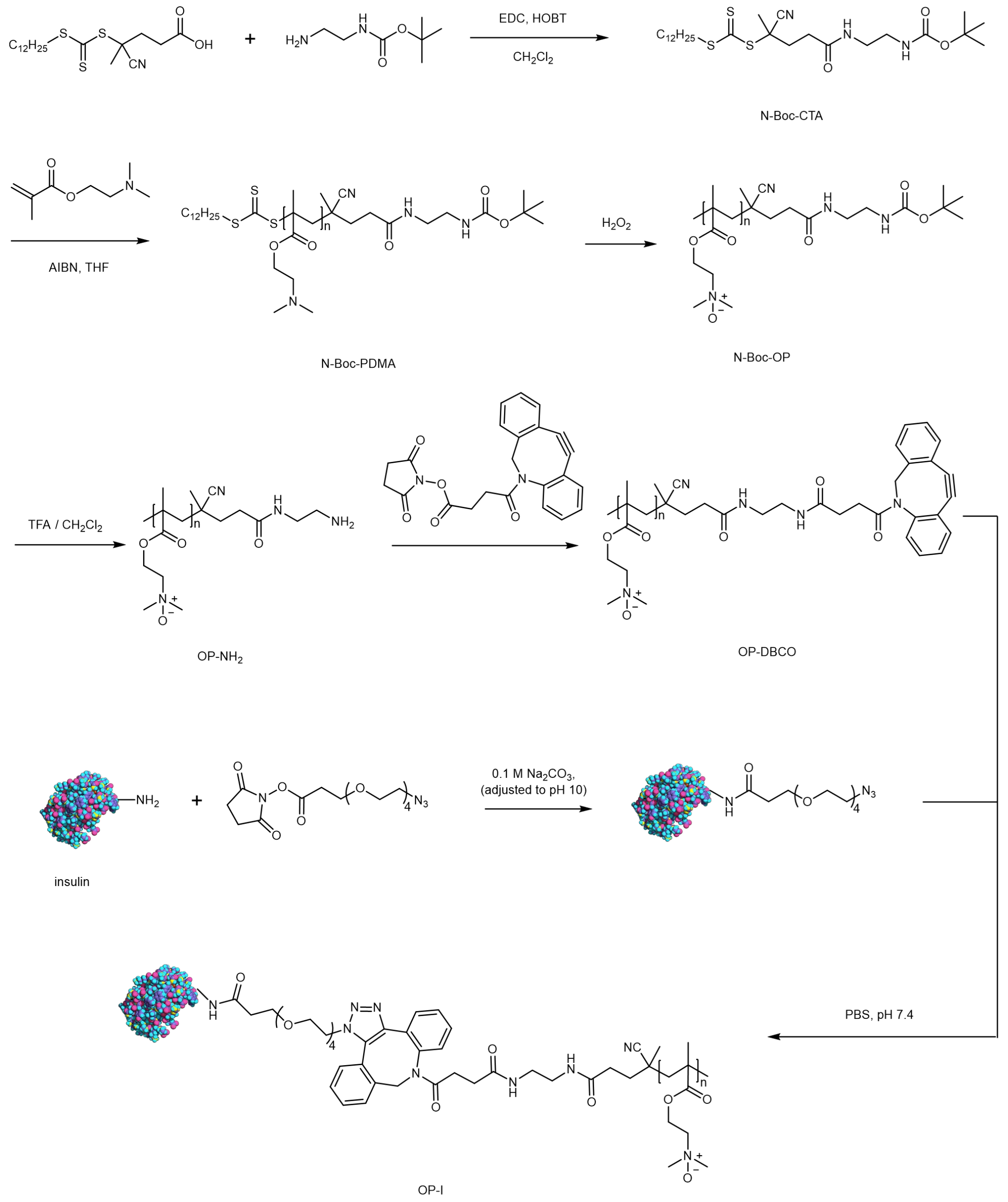

Supplementary Fig. 6 | The synthetic route to OP-I.

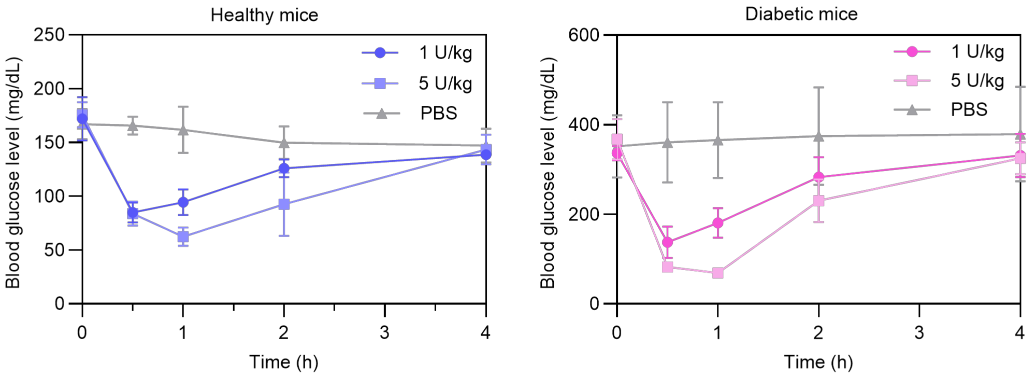

Supplementary Fig. 7 | Blood glucose levels (BGLs) of healthy mice and diabetic mice after subcutaneous (*s.c.*) injection of different doses of native insulin. Data are presented as mean ± s.d.; *n* = 5 mice.

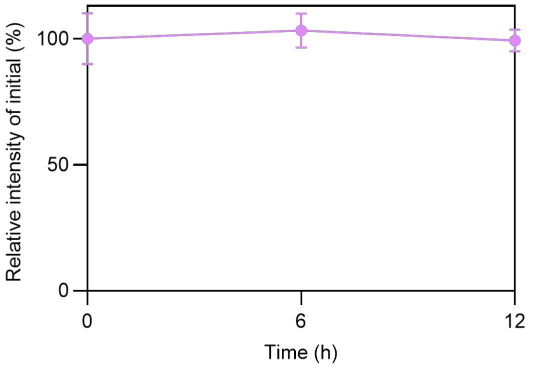

Supplementary Fig. 8 | HPLC analysis of the OP-I^{Cy5} stability in DMEM medium with 10% FBS. The peak area detected at 640 nm excitation/660 nm emission was normalised to the initial value. Data are presented as mean ± s.d.; *n* = 3 independent experiments.

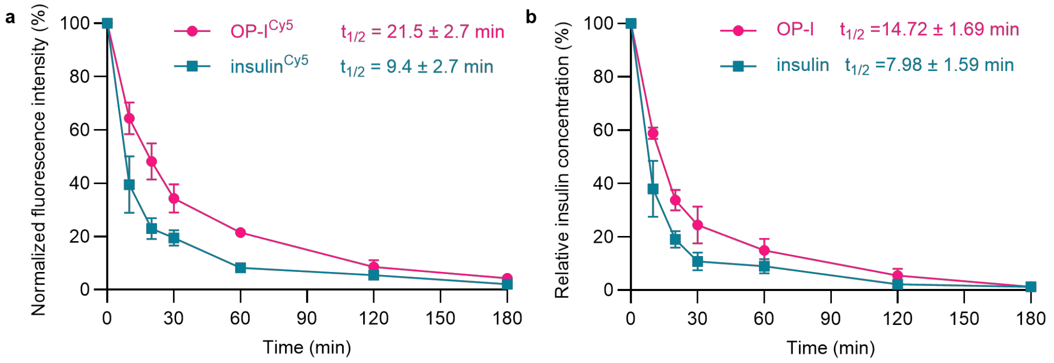

Supplementary Fig. 9 | Blood clearance kinetics of OP-I. a, Normalised blood clearance profiles, expressed as the percentage of remaining fluorescence intensity relative to that of the first sampling point as a function of time. OP-I^{Cy5} or insulin^{Cy5} was intravenously injected *via* the tail vein at a Cy5-eq dose of 0.1 mg/kg. **b,** Blood clearance profiles of insulin and OP-I analyzed by enzyme-linked immunosorbent assay (ELISA). OP-I or insulin (insulin-eq. dose: 1 U/kg) was intravenously injected *via* the tail vein. The insulin concentration relative to that of the first sampling point is shown as a function of time. Data are mean ± s.d.; n = 3 mice for panel a, n = 5 mice for panel b.

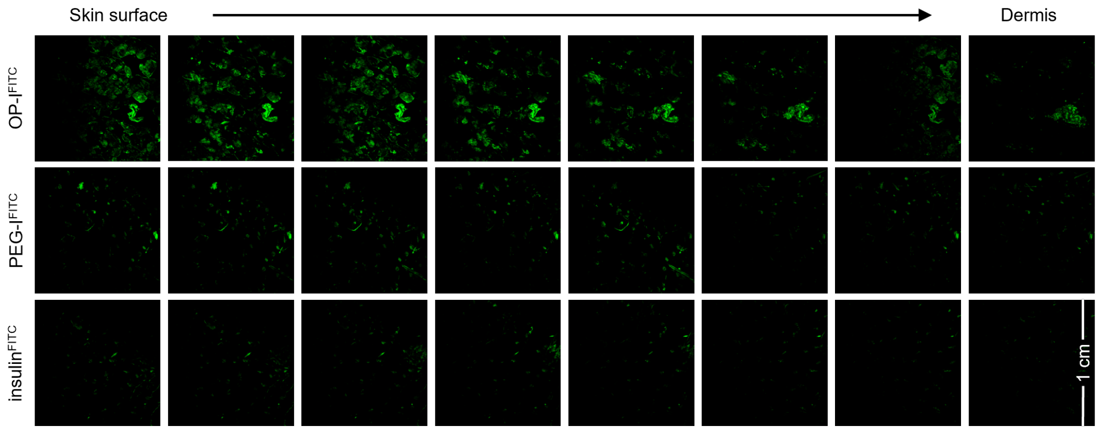

Supplementary Fig. 10 | Skin permeability of OP-I^{FITC} characterised by intravital two-photon microscopy. Sequential z-stack imaging of the mouse dorsal skin was performed after topical application for 4 h with FITC-labelled OP-I^{FITC}, PEG-I^{FITC}, or native insulin^{FITC} (FITC-eq. dose: 0.2 mL of 10 µg/mL; application area: 1.13 cm^2) using intravital two-photon microscopy. Z-step, 9 µm intervals. The images are representative of *n* = 3 independent experiments.

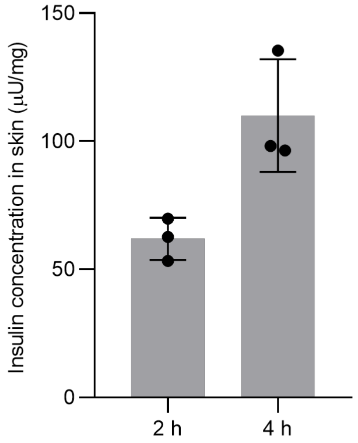

Supplementary Fig. 11 | The content of insulin in the skin following transdermal administration of OP-I. Insulin concentrations in the mouse dorsal skin at 2 h and 4 h post-topical application of OP-I were detected by ELISA (insulin-eq. dose: 116 U/kg; 0.2 mL of 0.5 mg/mL solution applied on 1.13 cm^2 dorsal skin). Data are mean ± s.d.; *n* = 3 mice.

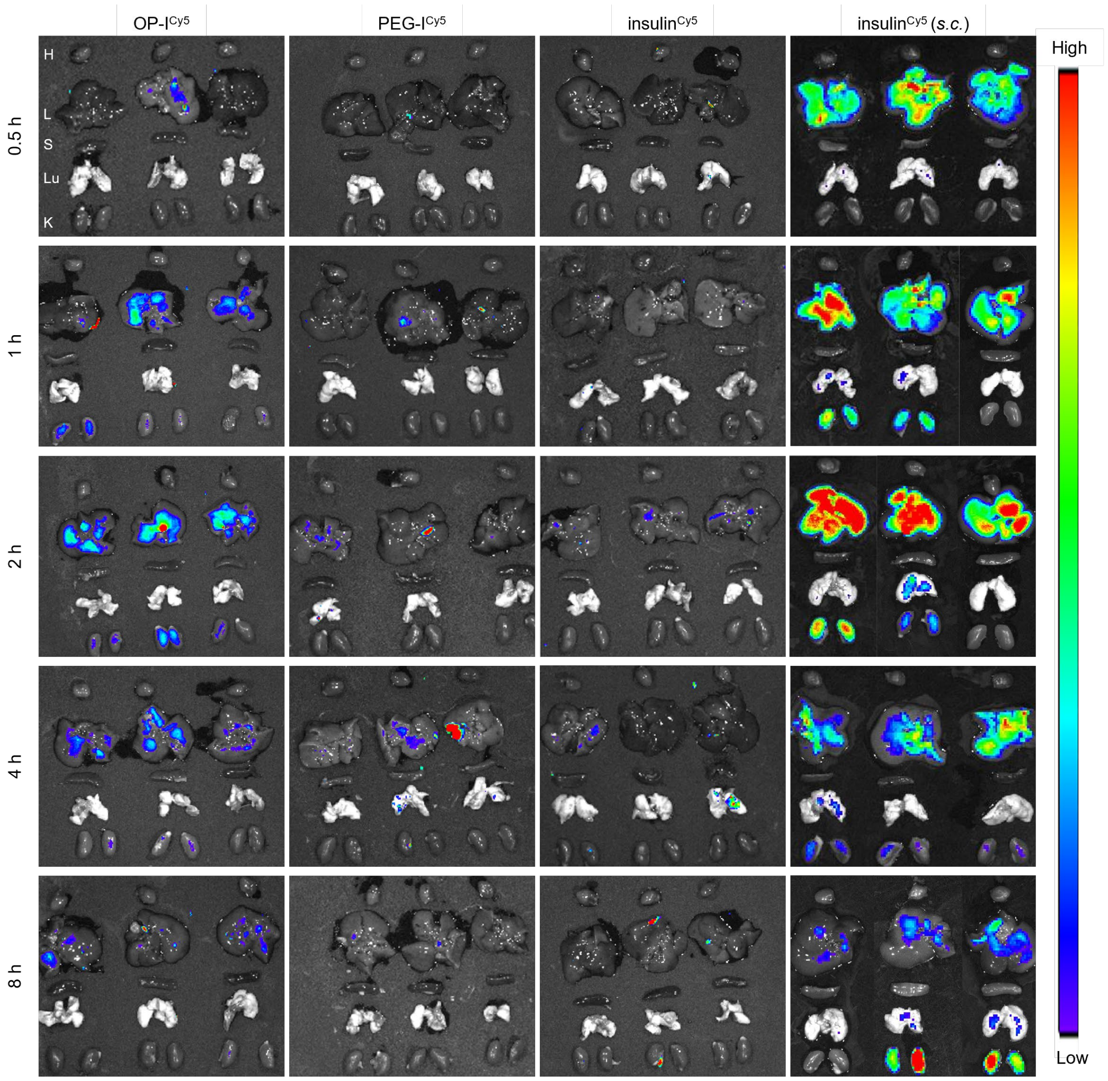

Supplementary Fig. 12 | Organ distribution of OP-I^{Cy5}. Fluorescence imaging of the major organs, including heart (H), liver (L), spleen (S), lungs (Lu), and kidneys (K), was performed after topical administration of OP-I^{Cy5}, PEG-I^{Cy5}, or insulin^{Cy5} solutions (Cy5-eq. dose: 0.2 mL of 10 µg/mL; application area: 1.13 cm^2) on the dorsal skin, or after *s.c.* injection of insulin^{Cy5} (Cy5-eq. dose: 50 µL of 25 µg/mL); n = 3 mice.

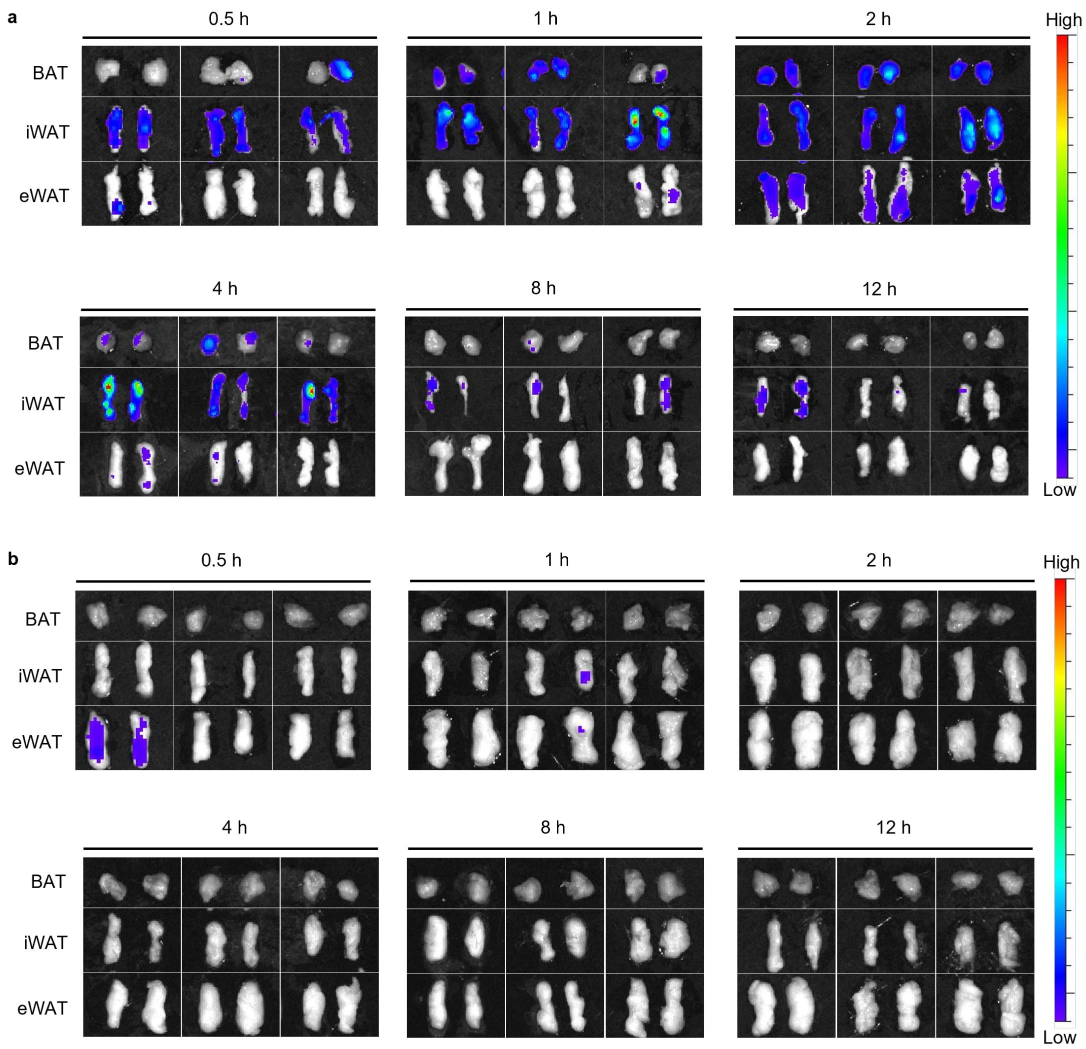

Supplementary Fig. 13 | Adipose distribution of OP-I^{Cy5}. Fluorescence imaging of mouse adipose tissues, including brown adipose (BAT), subcutaneous white adipose (iWAT), and visceral white adipose (eWAT), was performed after topical administration of OP-I^{Cy5} (Cy5-eq, dose: 0.2 mL of 10 µg/mL; application area: 1.13 cm^2) on the dorsal skin (a) or *s.c.* injection of insulin^{Cy5} (Cy5-eq. dose: 50 µL of 25 µg/mL) (b); *n* = 3 mice.

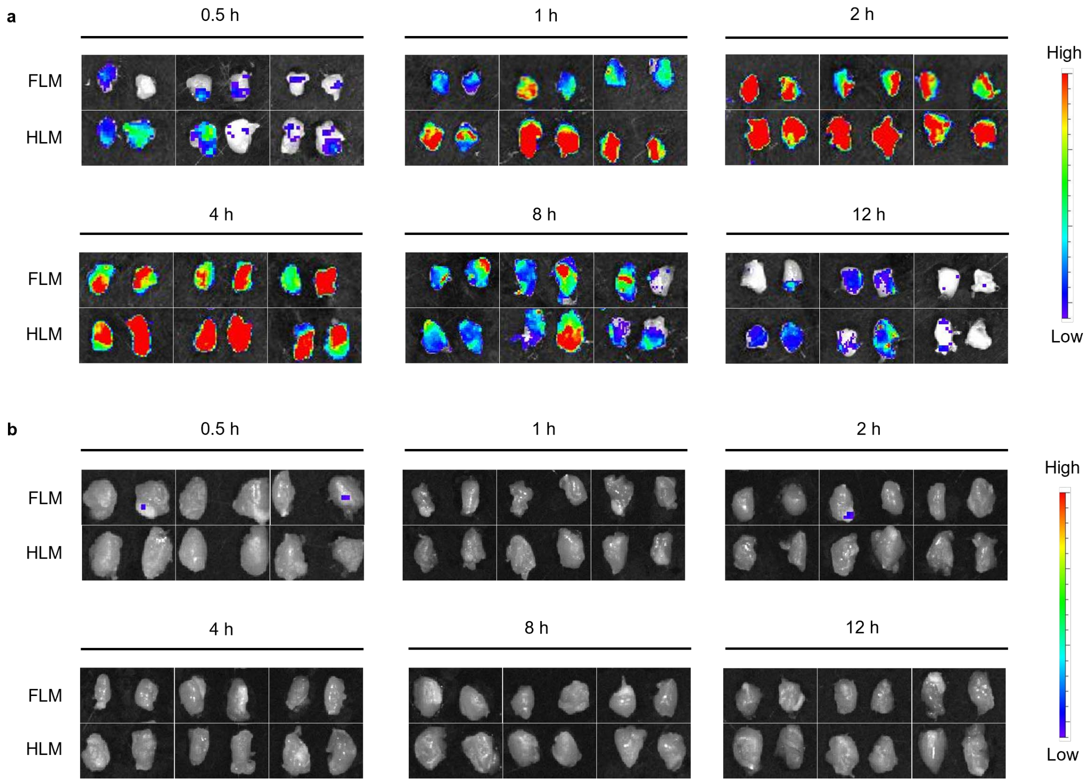

Supplementary Fig. 14 | Muscle distribution of OP-I^{Cy5}. Fluorescence imaging of the mouse forelimb muscle (FLM) and hindlimb muscle (HLM) was performed after topical administration of OP-I^{Cy5} (Cy5-eq, dose: 0.2 mL of 10 µg/mL; application area: 1.13 cm^2) on the dorsal skin (a) or *s.c.* injection of insulin^{Cy5} (Cy5-eq. dose: 50 µL of 25 µg/mL) (b); *n* = 3 mice.

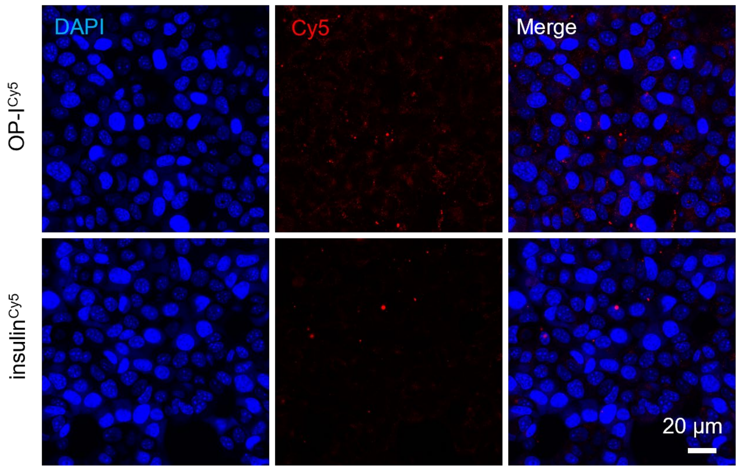

Supplementary Fig. 15 | Cellular uptake of OP-I^{Cy5} or insulin^{Cy5} (red) by AML-12 cells observed using CLSM. Cy5-eq. dose: 1 μg/mL; 4 h incubation. The images are representative of *n* = 3 independent experiments.

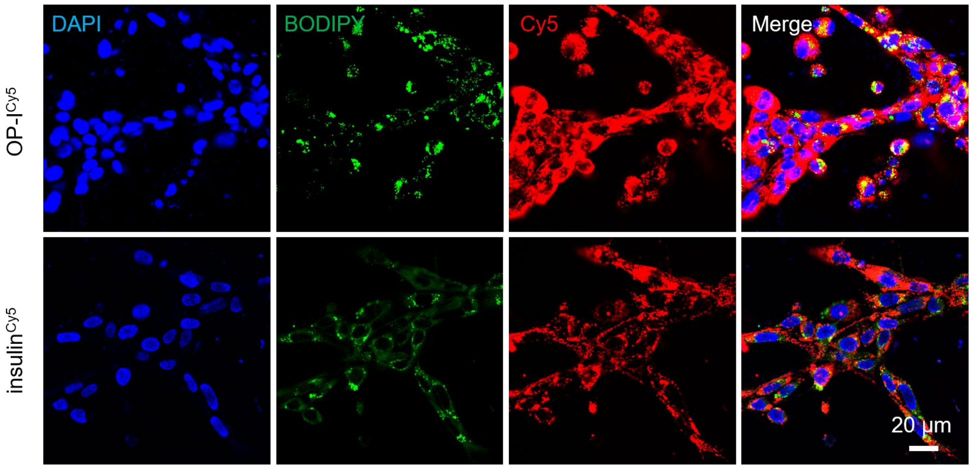

Supplementary Fig. 16 | Cellular uptake of OP-I^{Cy5} or insulin^{Cy5} (red) by 3T3-L1 differentiated adipocytes and their colocalization with adipocyte lipid droplets (green) observed using CLSM. Cy5-eq. dose: 1 μg/mL; 4 h incubation. The images are representative of *n* = 3 independent experiments.

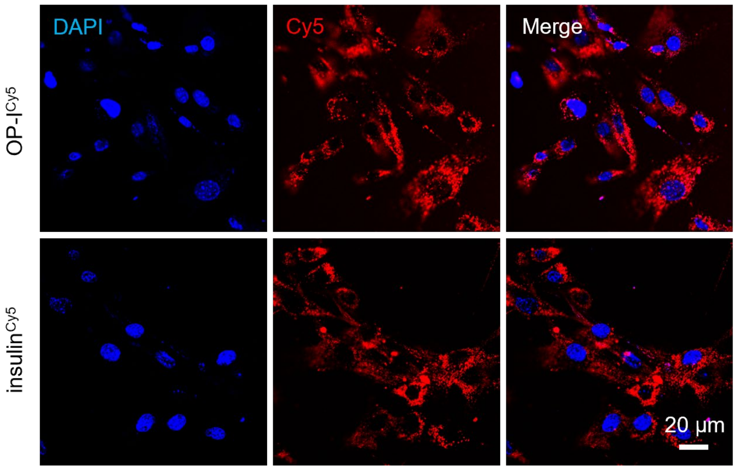

Supplementary Fig. 17 | Cellular uptake of OP-I^{Cy5} or insulin^{Cy5} (red) by mouse skeletal muscle cells observed using CLSM. Cy5-eq. dose: 1 μg/mL; 4 h incubation. The images are representative of *n* = 3 independent experiments.

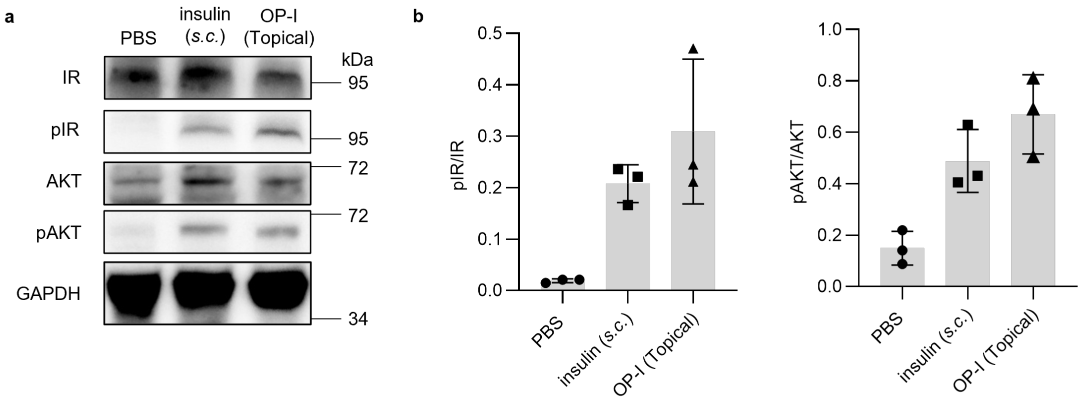

Supplementary Fig. 18 | Insulin receptor activation and signalling pathways of OP-I. a, Western blot analysis of phosphorylated IR (pIR) and phosphorylated AKT (pAKT) levels in the mouse skeletal muscle. Tissues were harvested from the STZ-induced diabetic mice 4 h after topical application of OP-I (insulin-eq. dose: 116 U/kg; 0.2 mL of 0.5 mg/mL solution applied on 1.13 cm^2 dorsal skin) or 1 h after *s.c.* injected with native insulin (5 U/kg) as a positive control. The blots are representative of *n* = 3 independent experiments. **b**, Quantification of pIR and pAKT levels. Data are mean ± s.d.; *n* = 3 mice.

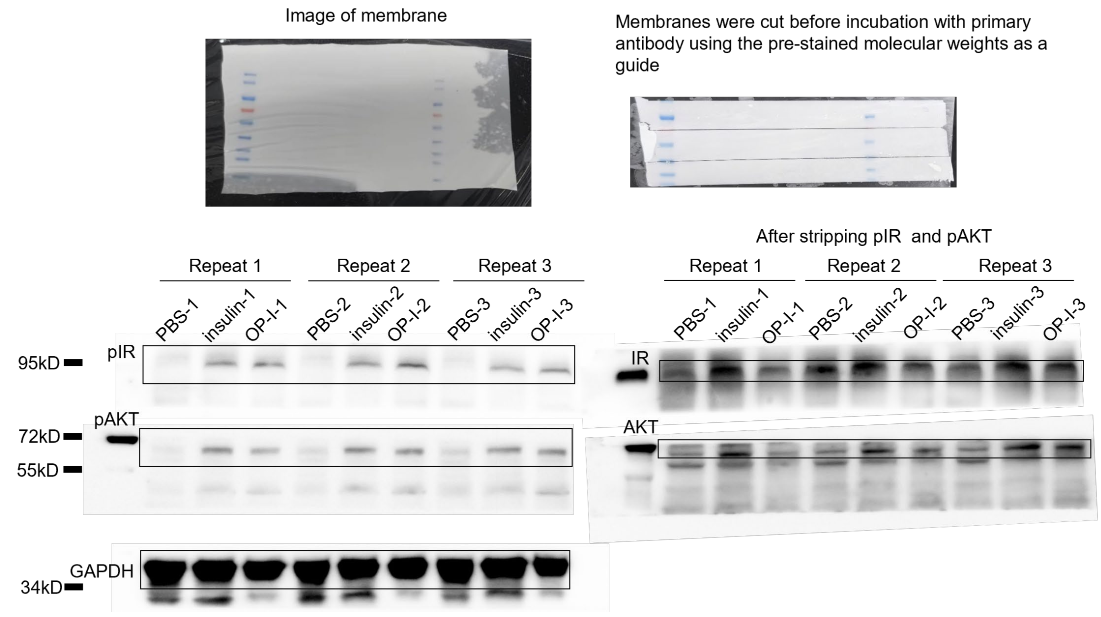

Supplementary Fig. 19 | The unprocessed blots for Supplementary Fig. 18.

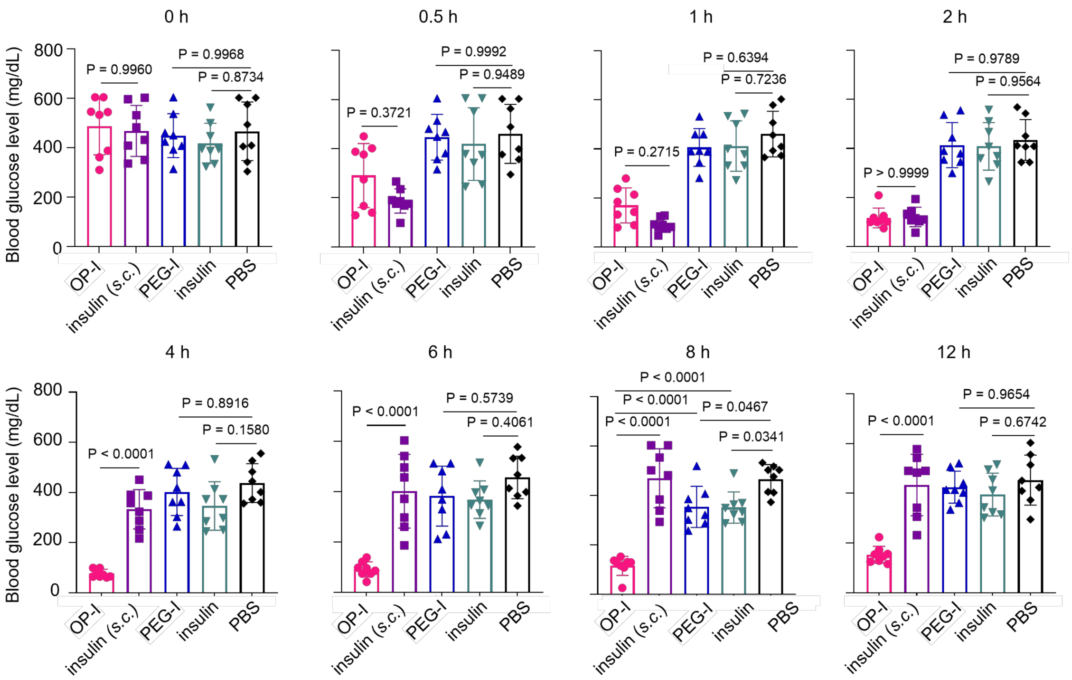

Supplementary Fig. 20 | BGLs of mice in each group at the time points shown in Fig. 3c. Data are mean ± s.d.; *n* = 8 mice. Significance was determined using a one-way ANOVA for multiple comparisons.

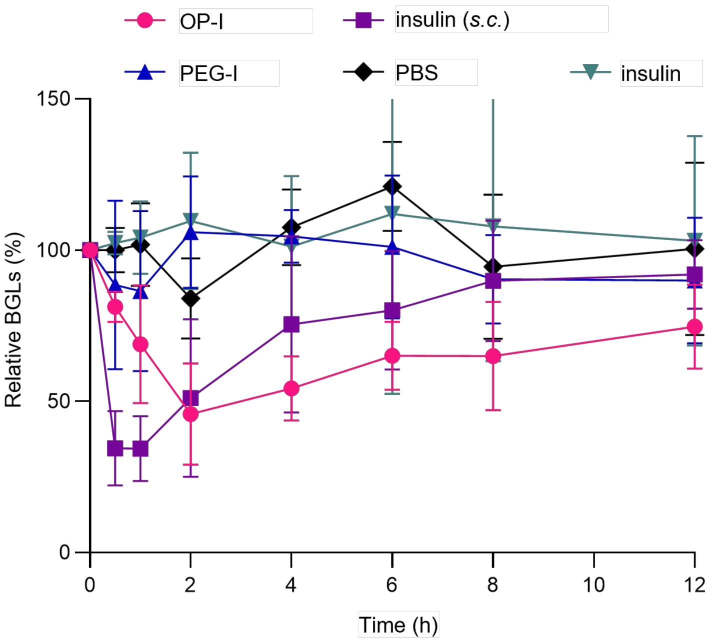

Supplementary Fig. 21 | Transdermal hypoglycemic effect of OP-I in healthy mice. BGLs of healthy mice were measured after topical administration of OP-I, PEG-I, or native insulin (insulin-eq. dose: 116 U/kg; 0.2 mL of 0.5 mg/mL; application area: 1.13 cm^2) on the dorsal skin, or *s.c.* injection of insulin (5 U/kg). Data are mean ± s.d.; *n* = 5 mice.

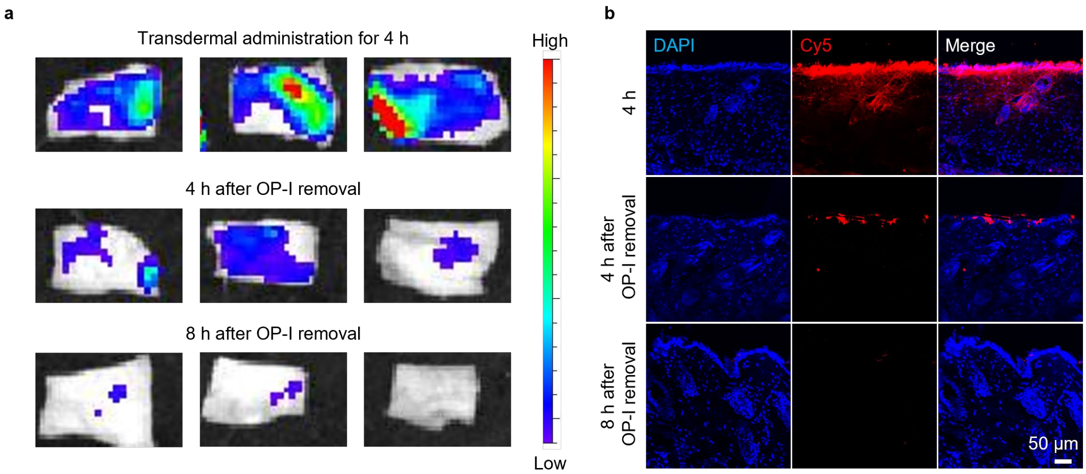

Supplementary Fig. 22 | Retention time of OP-I^{Cy5} in the skin. OP-I^{Cy5} in a diffusion cell (Cy5-eq. dose: 0.2 mL of 10 μg/mL; application area: 1.13 cm^2) was topically applied on the dorsal skin of C57BL/6J mice for 4 h and then removed; one group was sacrificed, another group was sacrificed after 4 h or 8 h for analysis. **a,** IVIS *ex vivo* imaging of the skin (*n* = 3 mice). **b,** CLSM imaging of the sections of the skin. The images are representative of *n* = 3 independent experiments.

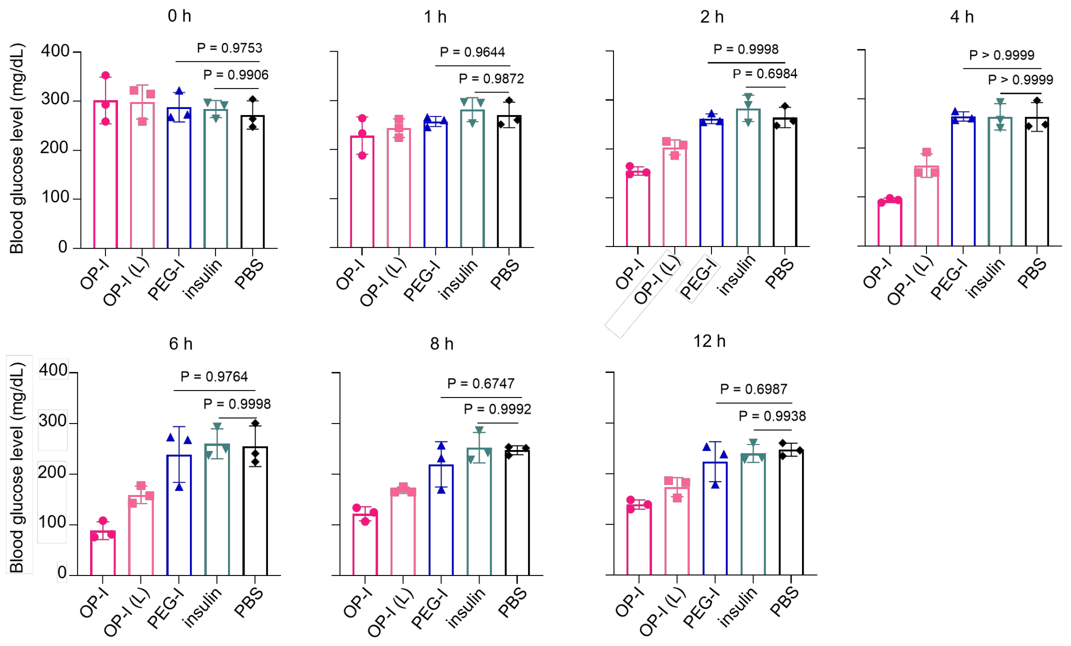

Supplementary Fig. 23 | BGLs of minipigs in each group at the timed points in Fig. 3i. Data are mean ± s.d.; *n* = 3 minipigs. Significance was determined using a one-way ANOVA for multiple comparisons.

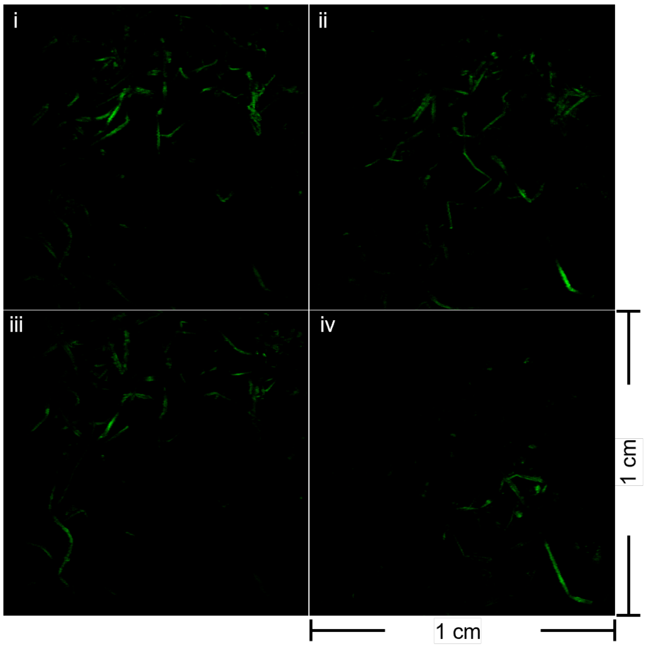

Supplementary Fig. 24 | Distribution of OP-I^{FITC} in SC. Sequential z-stack imaging of the mouse dorsal skin was performed after 4 h of topical application with OP-I^{FITC} (FITC-eq. dose: 0.2 mL of 10 µg/mL; application area: 1.13 cm^2), captured using intravital two-photon microscopy. The images are representative of *n* = 3 independent experiments.

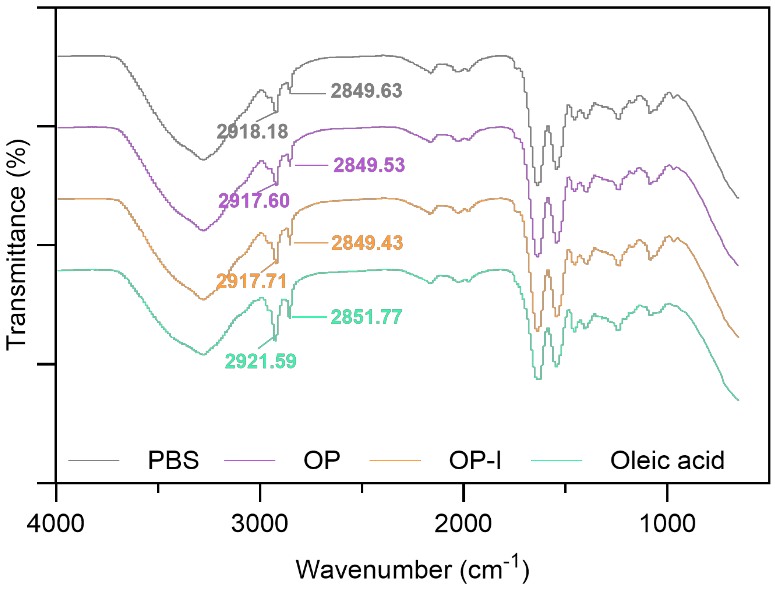

Supplementary Fig. 25 | SC lipid order of mouse skin after treatment with OP or OP-I. The Fourier transform infrared (FTIR) spectroscopy was performed on the mouse dorsal skin SC 24 h post-topical application of PBS, OP (0.23 mg/cm^2), OP-I (OP-eq. dose: 0.23 mg/cm^2)**,** or oleic acid (22.7 mg/cm^2) using a Franz diffusion cell system.

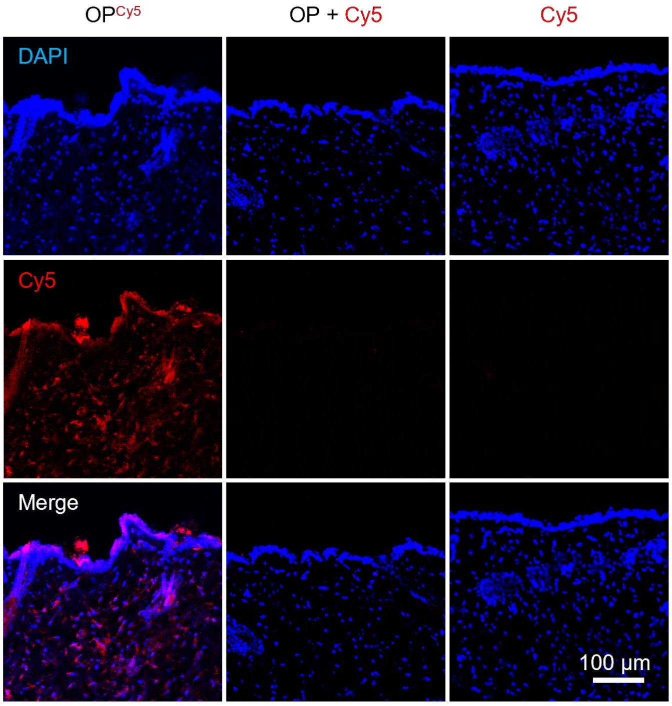

Supplementary Fig. 26 | Skin permeation of physical mixtures of OP with Cy5. The CLSM imaging of the mouse dorsal skin slices was performed after topical application for 4 h with OP^{Cy5}, a mixture of OP and Cy5, or free Cy5 (Cy5-eq. dose: 0.2 mL of 10 µg/mL; application area: 1.13 cm^2). The images are representative of n = 3 independent experiments.

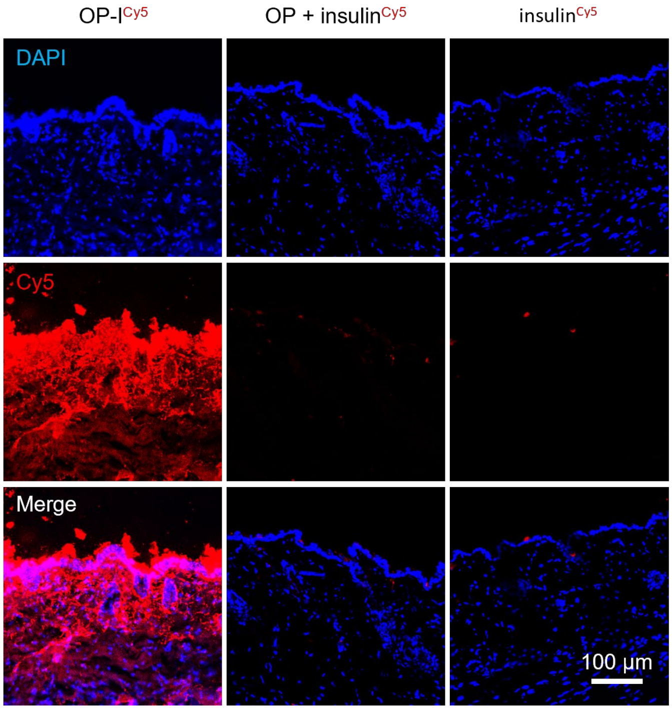

Supplementary Fig. 27 | Skin permeation of physical mixtures of OP with insulin^{Cy5}. The CLSM imaging of the mouse dorsal skin slices was performed after topical application for 4 h with OP-I^{Cy5}, a mixture of OP and insulin^{Cy5}, or insulin^{Cy5} (Cy5-eq. dose: 0.2 mL of 10 µg/mL; application area: 1.13 cm^2). The images are representative of *n* = 3 independent experiments.

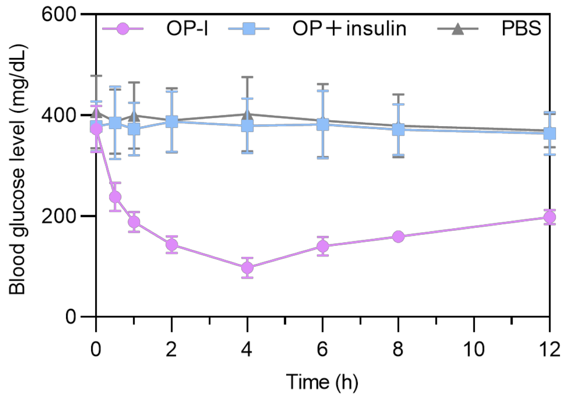

Supplementary Fig. 28 | Transdermal hypoglycemic effect of the physical mixture of OP with insulin. BGLs in the diabetic mice were measured after topical application of PBS, OP-I, or the mixture of OP and insulin (insulin-eq. dose: 116 U/kg; 0.2 mL of 0.5 mg/mL solution applied on 1.13 cm^2 dorsal skin). Data are mean ± s.d.; *n* = 5 mice.

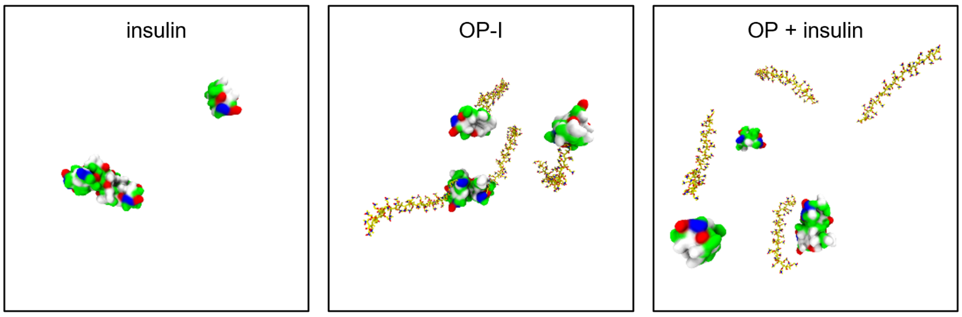

Supplementary Fig. 29 | Snapshots of 400-ns MD results with multiple molecules showing that OP did not bind insulin even though they carry opposite charges at pH 6.0.

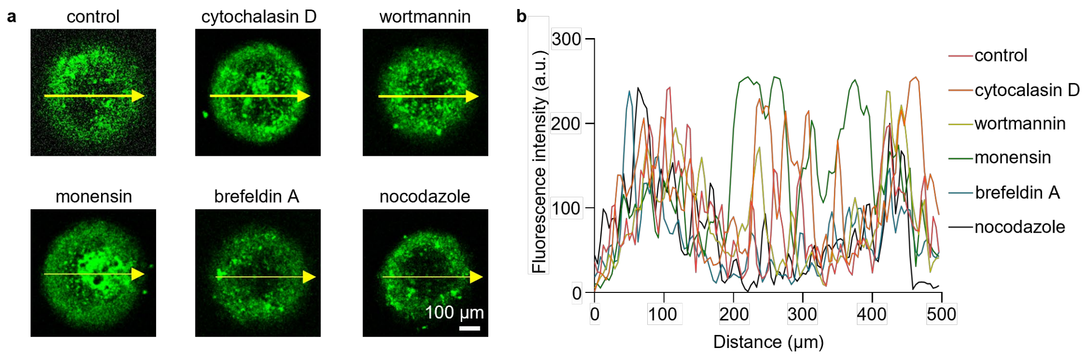

Supplementary Fig. 30 | Effects of transcytosis inhibitors on the permeability of OP-I^{FITC} in HaCat spheroids. a, CLSM imaging of the distribution of OP-I^{FITC} within HaCat spheroids. Spheroids were pre-treated with the indicated inhibitors for 4 h, followed by incubation with OP-I^{FITC} (FITC-eq. dose: 1 μg/mL) for 4 h. The images are representative of *n* = 3 independent experiments. **b**, Line-scan analysis of FITC fluorescence along a randomly selected line (yellow arrow in a).

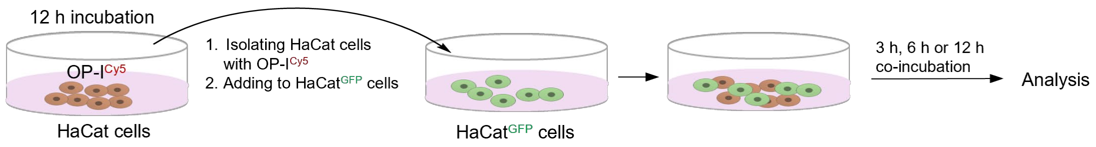

Supplementary Fig. 31 | Experimental illustration of cell-contact-dependent transfer of OP-I^{Cy5} from OP-I^{Cy5}-pretreated HaCat cells to untreated HaCat^{GFP} cells.

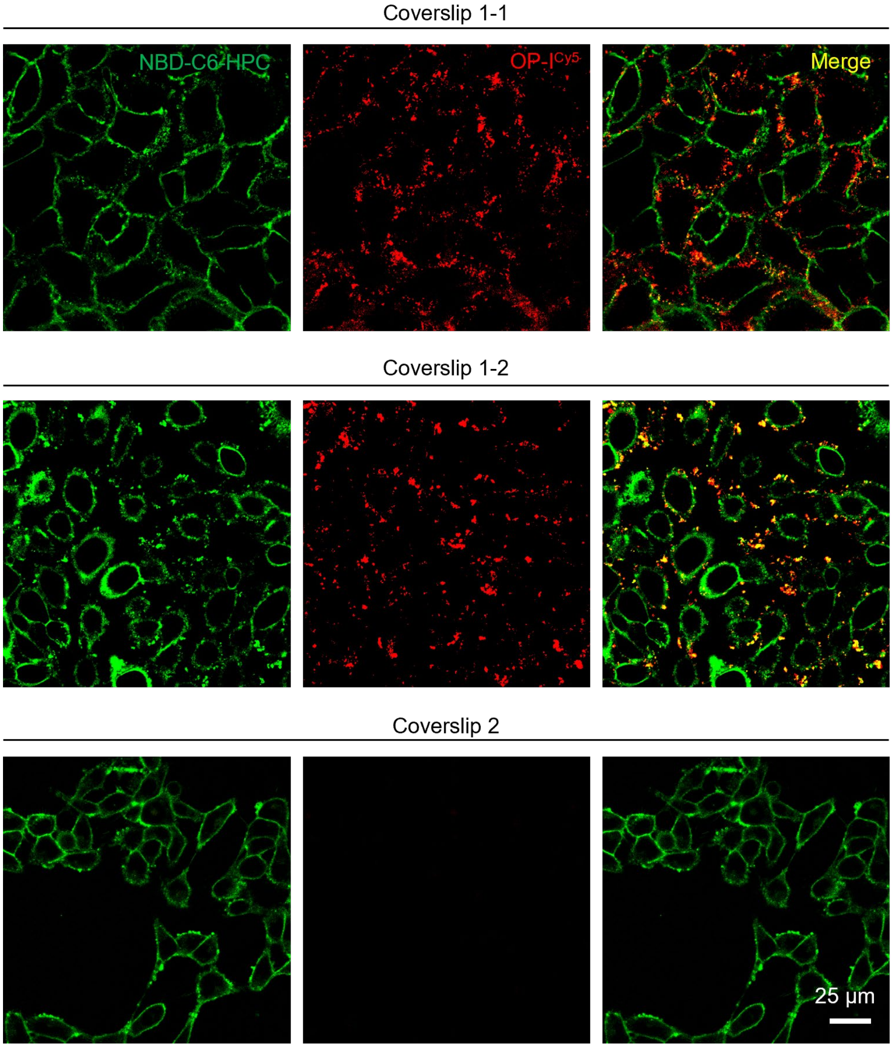

Supplementary Fig. 32 | Intercellular transfer of OP-I^{Cy5} among non-contact cells visualised by CLSM. HaCat cells (~10^5) on Coverslip 1 were cultured in a medium containing OP-I^{Cy5} (Cy5-eq. dose: 1 μg/mL) for 4 h and then rinsed and imaged (Coverslip 1-1). The Coverslip 1-1 was transferred into fresh culture medium with Coverslip 2 pre-seeded with HaCat cells, while both coverslips were not in contact. After 12 h of incubation, both Coverslip 1 (now noted as Coverslip 1-2) and Coverslip 2 were rinsed and imaged. Cell membranes were stained with NBD-C6-HPC (green). The images are representative of n = 3 independent experiments.

Supplementary Table 1 | Summary of skin permeation parameters of OP-I across the EpiKutis® model. Data are mean ± s.d.; n = 3 independent experiments. Significance compared to OP-I was determined using a two-tailed unpaired Student's t-test.

	Cumulative amount of insulin permeation per unit area in 24 h ($\mu g/cm^2$)	J_{ss} ($\mu g/cm^2/h$)	K_p (cm/h)
OP-I	14.50 ± 2.41	0.50 ± 0.12	2.94×10^{-3}
PEG-I	2.45 ± 0.05 (P = 0.0010)	0.10 ± 0.01 (P = 0.0040)	0.65×10^{-3}
insulin	1.47 ± 0.12 (P = 0.0007)	0.05 ± 0.01 (P = 0.0026)	0.32×10^{-3}

Supplementary Table 2 | Biosafety of OP-I following transdermal administration in minipigs. Blood biochemistry and blood routine examination of minipigs were performed after topical treatment of OP-I dispersed in W/O cream on the abdominal skin (insulin-eq. dose: 40 mL of 1 mg/mL, application area: 400 cm^2) as shown in Fig. 3i. Data are presented as mean ± s.d.; *n* = 3 minipigs. All values were within normal reference ranges.

Biochemical indicator		Blood routine	
Albumin (ALB) (g/L)	36.3 ± 0.3	Red blood cells (RBC) (10^{12}/L)	6.6 ± 1.0
Total protein (Tp) (g/L)	73.2 ± 0.2	Hemoglobin (HGB) (g/L)	122.6 ± 1.4
Globulin (GLO) (g/L)	36.0 ± 1.0	Mean red blood cell volume (MCV) (fL)	54.4 ± 1.4
Serum aspartate amino-transferase (AST) (U/L)	20.3 ± 2.1	Mean red blood cell hemoglobin concentration MCHC (g/L)	347.3 ± 4.0
Serum alkaline phosphatase (ALP) (U/L)	212.0 ± 1.0	Red blood cell distribution width (RDW) (%)	19.9 ± 1.9
