# Supplementary material for: A skin-permeable polymer for non-invasive transdermal insulin delivery
Source: Nature. 2025 Nov 19;648(8093):459–67. doi: 10.1038/s41586-025-09729-x (PMC12695667; doi:10.1038/s41586-025-09729-x)
Supplement: Supplementary file 2 — Reporting Summary [file 41586_2025_9729_MOESM2_ESM.pdf]

nature portfolio

Corresponding author(s): Youqing Shen; Ruhong Zhou; Rongjun Chen; Jiajia Xiang

Last updated by author(s): Sep 12, 2025

Reporting Summary

Nature Portfolio wishes to improve the reproducibility of the work that we publish. This form provides structure for consistency and transparency in reporting. For further information on Nature Portfolio policies, see our Editorial Policies and the Editorial Policy Checklist.

Statistics

For all statistical analyses, confirm that the following items are present in the figure legend, table legend, main text, or Methods section.

n/a	Confirmed	
☐	☒	The exact sample size (*n*) for each experimental group/condition, given as a discrete number and unit of measurement
☐	☒	A statement on whether measurements were taken from distinct samples or whether the same sample was measured repeatedly
☐	☒	The statistical test(s) used AND whether they are one- or two-sided<br>*Only common tests should be described solely by name; describe more complex techniques in the Methods section.*
☒	☐	A description of all covariates tested
☒	☐	A description of any assumptions or corrections, such as tests of normality and adjustment for multiple comparisons
☐	☒	A full description of the statistical parameters including central tendency (e.g. means) or other basic estimates (e.g. regression coefficient) AND variation (e.g. standard deviation) or associated estimates of uncertainty (e.g. confidence intervals)
☐	☒	For null hypothesis testing, the test statistic (e.g. *F*, *t*, *r*) with confidence intervals, effect sizes, degrees of freedom and *P* value noted<br>*Give P values as exact values whenever suitable.*
☒	☐	For Bayesian analysis, information on the choice of priors and Markov chain Monte Carlo settings
☒	☐	For hierarchical and complex designs, identification of the appropriate level for tests and full reporting of outcomes
☐	☒	Estimates of effect sizes (e.g. Cohen's *d*, Pearson's *r*), indicating how they were calculated

Our web collection on statistics for biologists contains articles on many of the points above.

Software and code

Policy information about availability of computer code

Data collection

GPC traces of samples were obtained by a Shimadzu Prominence Plus LC-20AD liquid chromatography equipped with two columns connected in series (PL aquagel-OH MIXED-H and PL aquagel-OH 30), a refractive index detector, and a UV/VIS detector.
RP-HPLC chromatography of samples were acquired by an Agilent 1260 Infinity II system equipped with a ZORBAX SB-C18 column (5 µm, 4.6 x 250 mm), and a 1260 infinity II ariable wavelength detector.
Far UV-CD spectra were recorded on a JASCO J-815 spectropolarimeter.
Mass spectrometry was obtained on a MALDI-TOF MS (Bruker Daltonics Inc.).
Fluorescence microscopy images were acquired by Nikon A1 confocal microscope, total internal reflection fluorescence microscopy (Olympus IX83) and Leica STELLARIS STED confocal microscope with 405-nm, 488-nm, and 640-nm lasers.
SEM images were obtained using a Hitichi SU-70 SEM.
STEM-HAADFI images and EDS-mapping were obtained using a STEM electron microscope (JEOL JEM-F200).
IVIS fluorescence images were acquired by the imaging system (IVIS Lumina XRMS Series III, PerkinElmer).
Two-photon fluorescence images were acquired by intravital two-photon microscopy platform (IVM-CMS model, IVIM Technology).
GROMACS (2020.6) was used to collect data from molecular dynamics simulations.
Flow cytometry data were obtained using a BD FACS Calibur and a CytoFLEX flow cytometer (Beckman Coulter).
Blood glucose data were collected by a blood glucose meter (Sinocare).
Blood routine data were collected using a Blood Cell Counter.
Biochemical indicator data were collected using an Automatic Biochemical Analyzer.
SPR experiments were performed on a Biacore X100 instrument (Cytiva).
Prep-HPLC was performed using a Waters Prep 150 LC system equipped with a Pursuit 5 C18 column (250 × 21.2 mm) and a Waters 2489 UV/Visible detector.
A Nano ZS Zetasizer (Malvern Instruments) was employed to measure zeta potentials.

Infrared spectrum was collected using a Thermo Scientific™ Nicolet™ iS50 FTIR spectrometer.
Western blot data were obtained by ChemiScope 3600 Mini Imaging System (Clinx Science Instruments Co., Ltd.).
Slices were collected by LEICA CM1950 cryostat (Leica Microsystems, Bannockburn, IL).
The ultrathin sections were prepared using a UC7 ultramicrotome (Leica Microsystems, Bannockburn, IL).
Fluorescence intensities and Optical Density were measured using a SpectraMax M2e microplate spectrophotometer (Molecular Devices).
CHARMM-GUI was used to build the SC lipid membrane in MD simulations.
CGMS15 (Dexcom G4 Platinum Continuous Glucose Monitor System, Dexcom) was used to measure blood glucose levels in minipigs.
Ex vivo transdermal experiments were performed using the Franz diffusion cell (RYJ-12B, Shanghai Huanghai Pharmaceutical Inspection Instrument Co., Ltd.).

Data analysis

Graphpad Prism (version 10.4.0) was used for data analysis.
Biacore X100 system control software (version 2.0.1.201) was used for SPR data acquisition.
FlowJo (version 10.0) was used for flow cytometry data analysis.
LabSolutions GPC (version 5.111) Software was used for the analysis of GPC data.
NIS-Elements Viewer (version 5.21), Leica Application Suite X (LAS X, version 3.7.5.24914), and Fiji ImageJ (version 2017) was used for fluorescence microscopy image analysis.
Living image®4.5.2 was used for IVIS fluorescence image analysis.
IVIM Studio (version 3.0509) was used for two-photon fluorescence image analysis.
GROMACS (version 2020.6) and VMD (version 1.9.4a51) were used for data analysis from MD simulations.
Omnic (version 9.2.0.41) was used for infrared spectrum analysis.
ChromScope Instrument Edition (version 2.1) was used to process prep-HPLC data.
Zetasizer Software (version 8.01.4906) was used to analyze DLS data.
Clinx ChemiCaptureMini (version 2.1.9.23) was used to analyze data from the chemiluminescence imager.
SoftMax Pro (version 5.4.6) was used to analyze microplate reader data.
OpenLab CDS ChemStation Edition (version C.01.07 SR3) was used to process RP-HPLC data.
DAS2 software (version 2.0) was used to analyze half-life.
The structures of OP and OP-I were constructed using Avogadro software (version 1.2).

For manuscripts utilizing custom algorithms or software that are central to the research but not yet described in published literature, software must be made available to editors and reviewers. We strongly encourage code deposition in a community repository (e.g. GitHub). See the Nature Portfolio guidelines for submitting code & software for further information.

Data

Policy information about availability of data

All manuscripts must include a data availability statement. This statement should provide the following information, where applicable:

- Accession codes, unique identifiers, or web links for publicly available datasets
- A description of any restrictions on data availability
- For clinical datasets or third party data, please ensure that the statement adheres to our policy

Data supporting the findings of this study are available in the Source Data file provided with this paper. The input files (.tpr files) for the key simulations in this study are available online (https://zenodo.org/records/17078486; accession code DOI 10.5281/zenodo.17078485).

Research involving human participants, their data, or biological material

Policy information about studies with human participants or human data. See also policy information about sex, gender (identity/presentation), and sexual orientation and race, ethnicity and racism.

Reporting on sex and gender	Human research participants are not applicable to this study.
Reporting on race, ethnicity, or other socially relevant groupings	Human research participants are not applicable to this study.
Population characteristics	Human research participants are not applicable to this study.
Recruitment	Human research participants are not applicable to this study.
Ethics oversight	Human research participants are not applicable to this study.

Note that full information on the approval of the study protocol must also be provided in the manuscript.

Field-specific reporting

Please select the one below that is the best fit for your research. If you are not sure, read the appropriate sections before making your selection.

☒ Life sciences ☐ Behavioural & social sciences ☐ Ecological, evolutionary & environmental sciences

For a reference copy of the document with all sections, see nature.com/documents/nr-reporting-summary-flat.pdf

Life sciences study design

All studies must disclose on these points even when the disclosure is negative.

Sample size	No sample size calculation was performed. The sample size was chosen based on previous experimental experience or rationals as follow. For in vivo experiments including skin permeability assay, tissue distribution assay, insulin biological half-life assay, lymphatic vessel co-localization assay, and western blot assay, we used at least n = 3 animals per group. Similar sample size was used in previous publications (Zhu, et al, (2024), Nat. Commun.). For in vivo studies in mice including subcutaneous glucose-lowering assay, transdermal glucose-lowering assay, and plasma insulin concentration detection assay, we used at least n = 5 mice per group. Similar sample size was used in previous publications (Han, et al, (2020), Nat. Nanotechnol.; Yu, et al, (2020), Nat. Biomed. Eng.; Yang, et al, (2022), Nat. Commun.). For transdermal glucose-lowering assay in diabetic minipigs, we used n = 3 minipigs. Similar sample size was used in previous publications (Zhang, et al, (2023), Nat. Nanotechnol.). For all in vitro experiments, we performed n = 3 independent experiments. Similar sample size was used in previous publications (Zhou, et al, (2019), Nat. Nanotechnol.; Chen, et al, (2021), Nat. Biomed. Eng.).
Data exclusions	There were no data exclusions except those resulting from technical errors making data interpretation impossible.
Replication	All experiments were successfully replicated with at least three biological replicates.
Randomization	All samples and animals were randomly allocated into experimental groups.
Blinding	Blinding was not performed in this study. All experiments used automated instruments and computational analysis, without subjective evaluations prone to investigator bias.

Behavioural & social sciences study design

All studies must disclose on these points even when the disclosure is negative.

Study description	*Briefly describe the study type including whether data are quantitative, qualitative, or mixed-methods (e.g. qualitative cross-sectional, quantitative experimental, mixed-methods case study).*
Research sample	*State the research sample (e.g. Harvard university undergraduates, villagers in rural India) and provide relevant demographic information (e.g. age, sex) and indicate whether the sample is representative. Provide a rationale for the study sample chosen. For studies involving existing datasets, please describe the dataset and source.*
Sampling strategy	*Describe the sampling procedure (e.g. random, snowball, stratified, convenience). Describe the statistical methods that were used to predetermine sample size OR if no sample-size calculation was performed, describe how sample sizes were chosen and provide a rationale for why these sample sizes are sufficient. For qualitative data, please indicate whether data saturation was considered, and what criteria were used to decide that no further sampling was needed.*
Data collection	*Provide details about the data collection procedure, including the instruments or devices used to record the data (e.g. pen and paper, computer, eye tracker, video or audio equipment) whether anyone was present besides the participant(s) and the researcher, and whether the researcher was blind to experimental condition and/or the study hypothesis during data collection.*
Timing	*Indicate the start and stop dates of data collection. If there is a gap between collection periods, state the dates for each sample cohort.*
Data exclusions	*If no data were excluded from the analyses, state so OR if data were excluded, provide the exact number of exclusions and the rationale behind them, indicating whether exclusion criteria were pre-established.*
Non-participation	*State how many participants dropped out/declined participation and the reason(s) given OR provide response rate OR state that no participants dropped out/declined participation.*
Randomization	*If participants were not allocated into experimental groups, state so OR describe how participants were allocated to groups, and if allocation was not random, describe how covariates were controlled.*

Ecological, evolutionary & environmental sciences study design

All studies must disclose on these points even when the disclosure is negative.

Study description	*Briefly describe the study. For quantitative data include treatment factors and interactions, design structure (e.g. factorial, nested, hierarchical), nature and number of experimental units and replicates.*
Research sample	*Describe the research sample (e.g. a group of tagged Passer domesticus, all Stenocereus thurberi within Organ Pipe Cactus National Monument), and provide a rationale for the sample choice. When relevant, describe the organism taxa, source, sex, age range and*

	any manipulations. State what population the sample is meant to represent when applicable. For studies involving existing datasets, describe the data and its source.
Sampling strategy	*Note the sampling procedure. Describe the statistical methods that were used to predetermine sample size OR if no sample-size calculation was performed, describe how sample sizes were chosen and provide a rationale for why these sample sizes are sufficient.*
Data collection	*Describe the data collection procedure, including who recorded the data and how.*
Timing and spatial scale	*Indicate the start and stop dates of data collection, noting the frequency and periodicity of sampling and providing a rationale for these choices. If there is a gap between collection periods, state the dates for each sample cohort. Specify the spatial scale from which the data are taken*
Data exclusions	*If no data were excluded from the analyses, state so OR if data were excluded, describe the exclusions and the rationale behind them, indicating whether exclusion criteria were pre-established.*
Reproducibility	*Describe the measures taken to verify the reproducibility of experimental findings. For each experiment, note whether any attempts to repeat the experiment failed OR state that all attempts to repeat the experiment were successful.*
Randomization	*Describe how samples/organisms/participants were allocated into groups. If allocation was not random, describe how covariates were controlled. If this is not relevant to your study, explain why.*
Blinding	*Describe the extent of blinding used during data acquisition and analysis. If blinding was not possible, describe why OR explain why blinding was not relevant to your study.*

Did the study involve field work? ☐ Yes ☐ No

Field work, collection and transport

Field conditions	*Describe the study conditions for field work, providing relevant parameters (e.g. temperature, rainfall).*
Location	*State the location of the sampling or experiment, providing relevant parameters (e.g. latitude and longitude, elevation, water depth).*
Access & import/export	*Describe the efforts you have made to access habitats and to collect and import/export your samples in a responsible manner and in compliance with local, national and international laws, noting any permits that were obtained (give the name of the issuing authority, the date of issue, and any identifying information).*
Disturbance	*Describe any disturbance caused by the study and how it was minimized.*

Reporting for specific materials, systems and methods

We require information from authors about some types of materials, experimental systems and methods used in many studies. Here, indicate whether each material, system or method listed is relevant to your study. If you are not sure if a list item applies to your research, read the appropriate section before selecting a response.

Materials & experimental systems

n/a	Involved in the study	
☐	☒	Antibodies
☐	☒	Eukaryotic cell lines
☒	☐	Palaeontology and archaeology
☐	☒	Animals and other organisms
☒	☐	Clinical data
☒	☐	Dual use research of concern
☒	☐	Plants

Methods

n/a	Involved in the study	
☒	☐	ChIP-seq
☐	☒	Flow cytometry
☒	☐	MRI-based neuroimaging

Antibodies

Antibodies used

LYVE1 Conjugated Antibody, Signalway Antibody (C49343-AF488)
Rabbit anti-phospho-AKT, Cell Signaling Technology (4056)
Rabbit anti-phospho-IR-IGF1R, Cell Signaling Technology (3024)
Mouse anti-GAPDH, Proteintech Group, Inc. (60004-1-Ig)
Rabbit anti-Insulin Receptor β, Cell Signaling Technology (3025)
Rabbit anti-AKT, Proteintech Group, Inc. (10176-2-AP)
Anti-histidine antibody, Cytiva Bio-technology Co., Ltd (28995056)
Secondary antibodies:

HRP-labeled Goat Anti-Rabbit IgG (H+L), Beyotime Biotechnology (A0208)
HRP-labeled Goat Anti-Mouse IgG (H+L), Beyotime Biotechnology (A0216)

Validation

All antibodies used were validated antibody suppliers per quality assurance as detailed on each supplier's website.
LYVE1 Conjugated Antibody: https://www.sabbiotech.com/products/C49343.
Rabbit anti-phospho-AKT (CST, 4056): https://www.cellsignal.cn/products/primary-antibodies/phospho-akt-thr308-244f9-rabbit-mab/4056.
Rabbit anti-phospho-IR-IGF1R (CST, 3024): https://www.cellsignal.cn/products/primary-antibodies/phospho-igf-i-receptor-b-tyr1135-1136-insulin-receptor-b-tyr1150-1151-19h7-rabbit-mab/3024.
Mouse anti-GAPDH (60004-1-Ig): https://ptgcn.com/products/GAPDH-Antibody-60004-1-Ig.htm#.
Rabbit anti-Insulin Receptor β (CST, 3025): https://www.cellsignal.cn/products/primary-antibodies/insulin-receptor-b-4b8-rabbit-mab/3025.
Rabbit anti-AKT (Proteintech, 10176-2-AP): https://ptgcn.com/products/AKT-Antibody-10176-2-AP.htm.
HRP-labeled Goat Anti-Rabbit IgG (H+L): https://www.beyotime.com/product/A0208.htm
HRP-labeled Goat Anti-Mouse IgG (H+L): https://www.beyotime.com/product/A0216.htm
Anti-histidine antibody: https://www.cytivalifesciences.com.cn/zh/cn/shop/protein-analysis/spr-label-free-analysis/spr-consumables/capture-reagents/his-capture-kit-p-05985.
Validated from manufacturer's website.

Eukaryotic cell lines

Policy information about cell lines and Sex and Gender in Research

Cell line source(s)

HaCat cell line was obtained from the Cell Bank of the Chinese Academy of Sciences (Shanghai, China). The cell expressing green fluorescence protein (HaCat-GFP cell) was established by lentivirus transfection of the GFP plasmids into HaCat cell according to the manufacturer’s protocol (Shanghai Genechem).
Mouse hepatoma cell line (AML-12), mouse embryonic fibroblast cell line (3T3-L1), and mouse skeletal muscle cell line (MSMC) were purchased from Shanghai Zhong Qiao Xin Zhou Biotechnology Co., Ltd.

Authentication

Cell lines have not been subjected to additional authentication.

Mycoplasma contamination

Cell lines have not been tested for mycoplasma contamination.

Commonly misidentified lines
(See ICLAC register)

No commonly misidentified cell lines were used.

Palaeontology and Archaeology

Specimen provenance

Provide provenance information for specimens and describe permits that were obtained for the work (including the name of the issuing authority, the date of issue, and any identifying information). Permits should encompass collection and, where applicable, export.

Specimen deposition

Indicate where the specimens have been deposited to permit free access by other researchers.

Dating methods

If new dates are provided, describe how they were obtained (e.g. collection, storage, sample pretreatment and measurement), where they were obtained (i.e. lab name), the calibration program and the protocol for quality assurance OR state that no new dates are provided.

☐ Tick this box to confirm that the raw and calibrated dates are available in the paper or in Supplementary Information.

Ethics oversight

Identify the organization(s) that approved or provided guidance on the study protocol, OR state that no ethical approval or guidance was required and explain why not.

Note that full information on the approval of the study protocol must also be provided in the manuscript.

Animals and other research organisms

Policy information about studies involving animals; ARRIVE guidelines recommended for reporting animal research, and Sex and Gender in Research

Laboratory animals

C57BL/6J mice, male, 6-8 weeks old, 25 g; SD rats, female, 8-12 weeks old, 200 g; Guangxi Bama-minipigs, male, 6 months old, 35-40 kg.

Wild animals

No wild animals were used in the study.

Reporting on sex

Male mice, male minipigs, and female rats were used in this study.

Field-collected samples

No field collected samples were used in the study.

Ethics oversight	All animal experiments were carried out according to the protocols approved by the Institutional Animal Care and Use Committee of Zhejiang University (Approval No. ZJU20250230).

Note that full information on the approval of the study protocol must also be provided in the manuscript.

Clinical data

Policy information about clinical studies

All manuscripts should comply with the ICMJE guidelines for publication of clinical research and a completed CONSORT checklist must be included with all submissions.

Clinical trial registration	*Provide the trial registration number from ClinicalTrials.gov or an equivalent agency.*
Study protocol	*Note where the full trial protocol can be accessed OR if not available, explain why.*
Data collection	*Describe the settings and locales of data collection, noting the time periods of recruitment and data collection.*
Outcomes	*Describe how you pre-defined primary and secondary outcome measures and how you assessed these measures.*

Dual use research of concern

Policy information about dual use research of concern

Hazards

Could the accidental, deliberate or reckless misuse of agents or technologies generated in the work, or the application of information presented in the manuscript, pose a threat to:

No	Yes	
☐	☐	Public health
☐	☐	National security
☐	☐	Crops and/or livestock
☐	☐	Ecosystems
☐	☐	Any other significant area

Experiments of concern

Does the work involve any of these experiments of concern:

No	Yes	
☐	☐	Demonstrate how to render a vaccine ineffective
☐	☐	Confer resistance to therapeutically useful antibiotics or antiviral agents
☐	☐	Enhance the virulence of a pathogen or render a nonpathogen virulent
☐	☐	Increase transmissibility of a pathogen
☐	☐	Alter the host range of a pathogen
☐	☐	Enable evasion of diagnostic/detection modalities
☐	☐	Enable the weaponization of a biological agent or toxin
☐	☐	Any other potentially harmful combination of experiments and agents

Plants

Seed stocks	This study does not include plants.
Novel plant genotypes	This study does not include plants.
Authentication	This study does not include plants.

ChIP-seq

Data deposition

☐ Confirm that both raw and final processed data have been deposited in a public database such as GEO.

☐ Confirm that you have deposited or provided access to graph files (e.g. BED files) for the called peaks.

Data access links<br>*May remain private before publication.*	*For "Initial submission" or "Revised version" documents, provide reviewer access links. For your "Final submission" document, provide a link to the deposited data.*
Files in database submission	*Provide a list of all files available in the database submission.*
Genome browser session<br>(e.g. UCSC)	*Provide a link to an anonymized genome browser session for "Initial submission" and "Revised version" documents only, to enable peer review. Write "no longer applicable" for "Final submission" documents.*

Methodology

Replicates	*Describe the experimental replicates, specifying number, type and replicate agreement.*
Sequencing depth	*Describe the sequencing depth for each experiment, providing the total number of reads, uniquely mapped reads, length of reads and whether they were paired- or single-end.*
Antibodies	*Describe the antibodies used for the ChIP-seq experiments; as applicable, provide supplier name, catalog number, clone name, and lot number.*
Peak calling parameters	*Specify the command line program and parameters used for read mapping and peak calling, including the ChIP, control and index files used.*
Data quality	*Describe the methods used to ensure data quality in full detail, including how many peaks are at FDR 5% and above 5-fold enrichment.*
Software	*Describe the software used to collect and analyze the ChIP-seq data. For custom code that has been deposited into a community repository, provide accession details.*

Flow Cytometry

Plots

Confirm that:

☒ The axis labels state the marker and fluorochrome used (e.g. CD4-FITC).

☒ The axis scales are clearly visible. Include numbers along axes only for bottom left plot of group (a 'group' is an analysis of identical markers).

☒ All plots are contour plots with outliers or pseudocolor plots.

☒ A numerical value for number of cells or percentage (with statistics) is provided.

Methodology

Sample preparation	For direct transfer experiment, the OP-ICy5 treated HaCat cells were extensively rinsed with sterilized PBS, isolated, and mixed with untreated HaCat-GFP cells at the same cell density. The mixed cells were co-cultured further 24 h. The cells were washed twice with PBS and analyzed by flow cytometry. Flow cytometry was performed on a BD FACS Calibur, and analysis was performed using the FlowJo software.
Instrument	Flow cytometry data were obtained using a BD FACS Calibur and a CytoFLEX flow cytometer (Beckman Coulter).
Software	Data analysis was performed using the FlowJo v10.0.
Cell population abundance	Cell sorting was not performed.
Gating strategy	First, an FSC-A vs. SSC-A gate was used to exclude debris and select for live cells based on their forward scatter (FSC) and side scatter (SSC) characteristics. Next, a GFP gate (FL1-H) was set to include cells that exhibit fluorescence in the GFP channel. Subsequently, a Cy5 gate (FL4-H) was established to isolate cells that are positive for Cy5 fluorescence. Finally, an intersection gate was applied between the GFP and Cy5 gates to identify cells that are positive for both GFP and Cy5, representing the dual-stained population.

☒ Tick this box to confirm that a figure exemplifying the gating strategy is provided in the Supplementary Information.

Magnetic resonance imaging

Experimental design

Design type

Indicate task or resting state; event-related or block design.

Design specifications

Specify the number of blocks, trials or experimental units per session and/or subject, and specify the length of each trial or block (if trials are blocked) and interval between trials.

Behavioral performance measures

State number and/or type of variables recorded (e.g. correct button press, response time) and what statistics were used to establish that the subjects were performing the task as expected (e.g. mean, range, and/or standard deviation across subjects).

Acquisition

Imaging type(s)

Specify: functional, structural, diffusion, perfusion.

Field strength

Specify in Tesla

Sequence & imaging parameters

Specify the pulse sequence type (gradient echo, spin echo, etc.), imaging type (EPI, spiral, etc.), field of view, matrix size, slice thickness, orientation and TE/TR/flip angle.

Area of acquisition

State whether a whole brain scan was used OR define the area of acquisition, describing how the region was determined.

Diffusion MRI ☐ Used ☐ Not used

Preprocessing

Preprocessing software

Provide detail on software version and revision number and on specific parameters (model/functions, brain extraction, segmentation, smoothing kernel size, etc.).

Normalization

If data were normalized/standardized, describe the approach(es): specify linear or non-linear and define image types used for transformation OR indicate that data were not normalized and explain rationale for lack of normalization.

Normalization template

Describe the template used for normalization/transformation, specifying subject space or group standardized space (e.g. original Talairach, MNI305, ICBM152) OR indicate that the data were not normalized.

Noise and artifact removal

Describe your procedure(s) for artifact and structured noise removal, specifying motion parameters, tissue signals and physiological signals (heart rate, respiration).

Volume censoring

Define your software and/or method and criteria for volume censoring, and state the extent of such censoring.

Statistical modeling & inference

Model type and settings

Specify type (mass univariate, multivariate, RSA, predictive, etc.) and describe essential details of the model at the first and second levels (e.g. fixed, random or mixed effects; drift or auto-correlation).

Effect(s) tested

Define precise effect in terms of the task or stimulus conditions instead of psychological concepts and indicate whether ANOVA or factorial designs were used.

Specify type of analysis: ☐ Whole brain ☐ ROI-based ☐ Both

Statistic type for inference

(See Eklund et al. 2016)

Specify voxel-wise or cluster-wise and report all relevant parameters for cluster-wise methods.

Correction

Describe the type of correction and how it is obtained for multiple comparisons (e.g. FWE, FDR, permutation or Monte Carlo).

Models & analysis

n/a	Involved in the study
☐	☐ Functional and/or effective connectivity
☐	☐ Graph analysis
☐	☐ Multivariate modeling or predictive analysis

Functional and/or effective connectivity

Report the measures of dependence used and the model details (e.g. Pearson correlation, partial correlation, mutual information).

Graph analysis

Report the dependent variable and connectivity measure, specifying weighted graph or binarized graph,

Graph analysis

subject- or group-level, and the global and/or node summaries used (e.g. clustering coefficient, efficiency, etc.).

Multivariate modeling and predictive analysis

Specify independent variables, features extraction and dimension reduction, model, training and evaluation metrics.
